# Pathway Analysis Based on a Genome-Wide Association Study of Polycystic Ovary Syndrome

**DOI:** 10.1371/journal.pone.0136609

**Published:** 2015-08-26

**Authors:** Unjin Shim, Han-Na Kim, Hyejin Lee, Jee-Young Oh, Yeon-Ah Sung, Hyung-Lae Kim

**Affiliations:** 1 Department of Internal Medicine, Seoul Seonam Hospital, Ewha Womans University Medical Center, Seoul, Korea; 2 Department of Biochemistry, Ewha Womans University School of Medicine, Seoul, Korea; 3 Department of Internal Medicine, Ewha Womans University School of Medicine, Seoul, Korea; Peiking University Third Hospital, CHINA

## Abstract

**Background:**

Polycystic ovary syndrome (PCOS) is one of the most common endocrine disorders in women of reproductive age, and it is affected by both environmental and genetic factors. Although the genetic component of PCOS is evident, studies aiming to identify susceptibility genes have shown controversial results. This study conducted a pathway-based analysis using a dataset obtained through a genome-wide association study (GWAS) to elucidate the biological pathways that contribute to PCOS susceptibility and the associated genes.

**Methods:**

We used GWAS data on 636,797 autosomal single nucleotide polymorphisms (SNPs) from 1,221 individuals (432 PCOS patients and 789 controls) for analysis. A pathway analysis was conducted using meta-analysis gene-set enrichment of variant associations (MAGENTA). Top-ranking pathways or gene sets associated with PCOS were identified, and significant genes within the pathways were analyzed.

**Results:**

The pathway analysis of the GWAS dataset identified significant pathways related to oocyte meiosis and the regulation of insulin secretion by acetylcholine and free fatty acids (all nominal gene-set enrichment analysis (GSEA) *P*-values < 0.05). In addition, *INS*, *GNAQ*, *STXBP1*, *PLCB3*, *PLCB2*, *SMC3* and *PLCZ1* were significant genes observed within the biological pathways (all gene *P*-values < 0.05).

**Conclusions:**

By applying MAGENTA pathway analysis to PCOS GWAS data, we identified significant pathways and candidate genes involved in PCOS. Our findings may provide new leads for understanding the mechanisms underlying the development of PCOS.

## Introduction

Polycystic ovary syndrome (PCOS) is one of the most common endocrine disorders in women of reproductive age, and it is characterized by chronic oligo-anovulation, clinical and/or biochemical hyperandrogenism and polycystic ovaries [1]. PCOS is a heterogeneous disorder with reproductive and metabolic phenotypes [2]. Studies have revealed family clustering in PCOS, suggesting genetic factors for the condition. Among first-degree relatives of women with PCOS, there is an increased prevalence of type 2 diabetes and androgen excess [3, 4]. In addition, the heritability of PCOS has been identified in a twin study [5].

Previous genetic studies on PCOS have been based mainly on candidate gene identification, which has revealed many of the genes involved in insulin expression and steroidogenesis [6, 7]. However, results for only a few of these genes have been confirmed through association studies, and the adopted approaches currently focus on the identification of susceptibility loci through genome-wide association studies (GWAS).

GWAS is a powerful, unbiased method for screening susceptible genes associated with complex diseases [8, 9]. The first GWAS of PCOS was performed in Han Chinese women and identified important susceptibility single nucleotide polymorphisms (SNPs) on chromosomes 2p16.3, 2p21 and 9p33.3; these SNPs were located in genes that included the thyroid adenoma-associated gene (*THADA*), *DENN/MADD* domain-containing 1A *(DENND1A)* and luteinizing hormone/choriogonadotropin receptor (*LHCGR*) [10]. In a study with a larger Han Chinese cohort, eight new loci were discovered [11]. In Korea, a GWAS of PCOS identified one novel locus with genome-wide significance on chromosome 8q24.2, located upstream of *KHDRBS3* (KH domain containing, RNA binding, signal transduction associated 3) associated with telomerase activity [12, 13].

One strength of GWAS is the ability to discover significant SNPs and novel genes associated with a disease. However, GWAS studies primarily focus on individual SNPs that meet a stringent significance criterion, neglecting the interplay of genes. Additionally, most identified SNPs lack functional relevance, explaining only a small portion of genetic heritability [14, 15]. The method also ignores the genetic interactions of complex diseases, and biological function cannot be determined. In GWAS, significant SNPs related to a certain disease may not be identified in other studies of the same disease because of their small effect size. To overcome this limitation, pathway-based approaches have been introduced and applied to GWAS datasets to further elucidate the pathogenesis of diseases [16].

The pathway-based approach integrates GWAS results with genes in biological pathways or gene sets from predefined human databases, ranking all genes according to their statistical significance [15, 17]. This method generates larger effect sizes, showing increased power to detect genes that may have been missed through GWAS, which improves the interpretability of genetic studies [17, 18]. In addition, because this approach can utilize genomic data to the maximal extent, unexpected or undetermined interactions of genes within a disease can also be identified. By applying pathway analysis to GWAS datasets, biological pathways associated with Crohn’s disease and common inflammatory pathways related to type 1 diabetes and rheumatoid arthritis can be discovered [19, 20].

Identifying the genetic pathways involved in PCOS may provide a more contextualized understanding of the mechanism underlying PCOS. The aim of this study was to use a pathway-based analysis of a GWAS dataset to elucidate the biological pathways involved in PCOS and the associated genes.

## Study Methods

### Subjects

The pathway analysis was conducted using a PCOS GWAS dataset that we generated previously. The dataset included data from 1,000 patients with PCOS and 1,000 controls. This study was performed in the Endocrinology and Gynecology Clinics of Ewha Womans University Hospital from December 2008 through November 2010. PCOS was diagnosed using the National Institutes of Health (NIH) criteria, which define the disorder as the presence of chronic oligo-anovulation and clinical and/or biochemical hyperandrogenism; the NIH criteria exclude other disorders, such as Cushing syndrome, adult-onset congenital adrenal hyperplasia and androgen secreting neoplasm [21]. Oligo-anovulation was defined as fewer than eight menstrual cycles per year. Biochemical hyperandrogenemia was defined as a total or free testosterone level above the 95^th^ percentile (total testosterone ≥ 67 ng/dL or free testosterone ≥ 0.84 ng/dL) based on the testosterone levels recorded in 1,120 healthy, regularly cycling women [22]. Clinical hyperandrogenism was evaluated based on the presence of hirsutism, defined as a modified Ferriman-Gallwey (mFG) score of 3 or above, which is the cutoff value for East Asian women recommended by the Androgen Excess and Polycystic Ovary Syndrome Society [23, 24].

### Anthropometric, biochemical and hormonal measurements

Weight and height were measured in all subjects, and body mass index (BMI) was calculated (kg/m^2^). Waist circumference was measured to the nearest 0.1 cm on bare skin during mid-respiration at the narrowest indentation between the tenth rib and the iliac crest. Systolic and diastolic blood pressures were also measured. Hirsutism was assessed by a single trained nurse using the mFG scoring method.

After an overnight fast of at least 8 hours, a venous blood sample was obtained from each subject on the third day of the follicular phase of the menstrual cycle. Standardized enzymatic methods were used to analyze lipid profiles, including serum total cholesterol, high-density lipoprotein (HDL) cholesterol and triglyceride levels. For evaluation of glucose tolerance, a standard 75 g oral glucose tolerance test (OGTT) was performed in all subjects after an overnight fast to determine fasting plasma glucose and 2-hour post-load glucose. Total testosterone levels were measured using the chemiluminescent immunoassay method (commercial kit, Siemens, New York, NY, USA), and sex hormone-binding globulin (SHBG) levels were measured using immunoradiometric assays (commercial kit, Diagnostic Products Corporation, Los Angeles, CA, USA). Using the formula from the International Society for the Study of the Aging Male (http://www.issam.ch/freetestos.htm), free testosterone levels were calculated using total testosterone, SHBG and albumin levels [22].

The institutional review board of Ewha Womans University Mokdong Hospital approved the study. Written informed consent was obtained from all participants.

### GWAS dataset analyses

Genomic DNA was extracted from individual peripheral blood samples and genotyped in 2,000 samples using the Illumina HumanOmni1-Quad v1 BeadChip (Illumina Inc., San Diego, CA, USA). Quality control (QC) procedures were applied using PLINK version 1.07 [25], excluding the samples through the following properties: genotyping calls < 95%, heterozygosity > 30%, markers with high missing call rate > 1%, minor allele frequency < 0.05 and significant deviation from Hardy-Weinberg equilibrium < 1 x 10^−6^. A total of 636,797 autosomal SNPs representing 1,922 individuals were obtained after the QC procedures. After excluding individuals with PCOS who did not satisfy the NIH diagnostic criteria, the data from 1,221 individuals (432 women with PCOS and 789 controls) were available. Additive models were used for analysis.

### Pathway-based analysis

A pathway analysis was conducted using meta-analysis gene-set enrichment of variant associations (MAGENTA) (http://broadinstitute.org/mpg/magenta) to identify biological pathways or gene sets associated with PCOS [26]. MAGENTA implements gene-set enrichment analysis (GSEA) associated with GWAS data through pathway annotations from the Gene Ontology (GO), Kyoto Encyclopedia of Genes and Genomes (KEGG), Protein Analysis Through Evolutionary Relationships (PANTHER), BioCarta and Reactome databases, which are web-based databases included in the Pathguide (http://pathguide.org) online resource.

The analytical steps of MAGENTA are as follows [26]. SNP association *P*-values and chromosome positions obtained from GWAS are mapped with genes that are located at a predetermined boundary. Gene scoring based on regional SNP *P*-values is completed, and SNPs with the most significant *P*-value within the predefined boundary (called the “best SNP *P*-value”) are selected. Gene scores are then corrected for confounders, including gene size, SNP numbers and linkage disequilibrium-related properties. Gene sets enriched with highly ranked gene scores are analyzed with the selected biological pathway or gene sets, and gene-set enrichment *P*-values are calculated. Additional information, including the 95^th^ and 75^th^ percentile cutoffs, the names of genes within each pathway or gene set, and the nominal GSEA *P*-value and false discovery rate (FDR), is analyzed through multiple test correction. Because the 75^th^ percentile cutoff demonstrates greater power in interpreting complex diseases with high polygenesis, we used this cutoff value for interpretation [26, 27]. After identifying the top-ranking biological pathways or gene sets associated with PCOS, significant genes that were observed within the identified pathways were further analyzed. Genes showing *P*-values of less than 0.05 were considered to be significant genes involved in the selected pathways or gene sets.

## Results

The clinical and biochemical characteristics of the women with PCOS and controls included in this study are shown in Table 1. The women with PCOS were younger than the controls, and their metabolic profiles, including BMI, waist circumference, systolic and diastolic blood pressure, total cholesterol, triglycerides, and fasting and post-load 2-hour glucose levels, were higher compared with the controls.

**Table 1:** Clinical and biochemical characteristics of the women with polycystic ovary syndrome and controls included in this study.

Characteristc	PCOS (n = 432)	Controls (n = 789)	*P*-value
Age (years)	24 ± 5	26 ± 4	< 0.001
Body mass index (kg/m^2^)	24.0 ± 4.7	21.0 ± 2.6	< 0.001
Waist circumference (cm)	79.2 ± 11.4	72.2 ± 7.0	< 0.001
Systolic blood pressure (mmHg)	110.8 ± 10.4	106.9 ± 9.0	< 0.001
Diastolic blood pressure (mmHg)	71.9 ± 9.0	69.4 ± 7.8	< 0.001
mFG score	2 ± 2	1 ± 1	< 0.001
Total testosterone (ng/dL)	76.7 ± 18.6	46.4 ± 15.6	< 0.001
Free testosterone (ng/dL)	1.20 ± 0.43	0.40 ± 0.19	< 0.001
Total cholesterol (mg/dL)	184.2 ± 30.6	174.0 ± 27.9	< 0.001
Triglycerides (mg/dL)	100.1 ± 62.4	73.7 ± 34.9	< 0.001
HDL-cholesterol (mg/dL)	49.5 ± 13.2	50.0 ± 10.6	0.492
Fasting plasma glucose (mg/dL)	87.4 ± 15.8	85.1 ± 6.8	0.006
Post-load 2-hour glucose (mg/dL)	112.3 ± 39.6	95.2 ± 18.5	< 0.001

Data are presented as means ± standard deviations. HDL, high-density lipoprotein; mFG, modified Ferriman-Gallwey.

The top ten significant biological pathways or gene sets associated with PCOS are displayed in Table 2. Pathways related to ovulation and insulin secretion, including oocyte meiosis (KEGG), the regulation of insulin secretion by acetylcholine (ACh) (Reactome) and the regulation of insulin secretion by free fatty acids (FFAs) (Reactome), were the top-ranking pathways associated with PCOS. Other pathways were also identified (all nominal GSEA *P*-values < 0.05), including neural tube closure (GO term), other kinases (PANTHER), the calcium signaling pathway (KEGG), acyltransferase (PANTHER), the negative regulation of osteoclast differentiation (GO term), cytoskeletal protein binding (GO term) and developmental processes (PANTHER). The FDR values for oocyte meiosis, the regulation of insulin secretion by ACh and FFAs and calcium signaling pathways were 0.078, 0.152, 0.110 and 0.222, respectively.

**Table 2:** Top ten significant biological pathways or gene sets associated with polycystic ovary syndrome.

Database	Biological pathway or gene set	95% cutoff (Top 5%)				75% cutoff (Top 25%)			
		Nominal GSEA P-value	FDR	Expected # of genes	Observed # of genes	Nominal GSEA P-value	FDR	Expected # of genes	Observed # of genes
KEGG	Oocyte meiosis	2.67E-1	1.000	5	7	5.00E-4	0.078	26	42
Reactome	Regulation of insulin secretion by acetylcholine	1.92E-2	0.705	1	4	7.00E-4	0.152	6	13
GO term	Neural tube closure	1.33E-1	0.733	1	3	1.10E-3	1.000	6	14
Reactome	Regulation of insulin secretion by free fatty acids	1.56E-2	0.725	1	4	1.50E-3	0.110	5	12
PANTHER	Other kinase	1.72E-1	1.000	2	4	2.00E-3	0.336	11	20
KEGG	Calcium signaling pathway	1.27E-1	1.000	8	12	2.10E-3	0.222	42	58
PANTHER	Acyltransferase	9.00E-4	0.236	5	13	2.60E-3	0.212	24	37
GO Term	Negative regulation of osteoclast differentiation	1.77E-2	0.538	1	3	2.90E-3	0.901	3	8
GO Term	Cytoskeletal protein binding	3.05E-1	0.791	2	3	2.90E-3	1.000	10	18
PANTHER	Developmental processes	1.22E-1	1.000	22	28	3.20E-3	0.761	112	137

FDR, false discovery rate; GO, gene ontology; GSEA, gene-set enrichment analysis; KEGG, Kyoto Encyclopedia of Genes and Genomes; PANTHER, Protein Analysis Through Evolutionary Relationships.

The genes involved in the biological pathways were further evaluated. The significant genes involved in the pathway of oocyte meiosis were *SMC3* (structural maintenance of chromosome 3), *CCNE2* (cyclin E2), *PPP2R5D* (protein phosphatase 2, regulatory subunit B, delta), *INS* (insulin), *PPP2R5C* (protein phosphatase 2, regulatory subunit B, gamma), *PLCZ1* (phospholipase C, zeta 1), *PPP2R5A* (protein phosphatase 2, regulatory subunit B, alpha), *PPP1CB* (protein phosphatase 1, catalytic subunit, beta isozyme) and *SPDYA* (speedy/RINGO cell cycle regulator family member A) (all gene *P*-values < 0.05). The genes *INS*, *STXBP1* (syntaxin binding protein 1), *PLCB3* (phospholipase C, beta 3), *GNAQ* (guanine nucleotide binding protein, q polypeptide) and *PLCB2* (phospholipase C, beta 2) were identified in the pathways related to the regulation of insulin secretion by ACh and the regulation of insulin secretion by FFAs (all gene *P*-values < 0.05) (Table 3). In the calcium signaling pathway, genes such as *LHCGR*, *PLCB3*, *PLCZ1*, *GNAQ*, *EGFR* (epidermal growth factor receptor) and *PLCB2* were significant. Detailed information on the genes identified in other biological pathways is shown in S1 Table. All pathway information was downloaded from the Pathguide online resource.

**Table 3:** Significant genes within the biological pathways or gene sets associated with polycystic ovary syndrome.

Biological pathway or gene set (database)	Genes (*P* <0.05)	Best SNP	CHR	SNP *P-*value
Oocyte meiosis (KEGG)	*SMC3*	rs3828047	10	7.85E-05
	*CCNE2*	rs4364818	8	2.96E-03
	*PPP2R5D*	rs1091000	6	3.00E-03
	*INS*	rs4648808	11	1.07E-03
	*PPP2R5C*	rs1274976	14	3.62E-03
	*PLCZ1*	rs1075339	12	5.55E-03
	*PPP2R5A*	rs1112082	1	8.54E-03
	*PPP1CB*	rs1158747	2	9.83E-03
	*SPDYA*	rs161811	2	1.01E-02
Regulation of insulin secretion by acetylcholine (REACTOME)	*INS*	rs4648808	11	1.07E-03
	*STXBP1*	rs1870516	9	5.80E-03
	*PLCB3*	rs1908490	11	4.02E-03
	*GNAQ*	rs7539775	9	2.30E-03
	*PLCB2*	rs2029490	15	4.11E-03
Regulation of insulin secretion by free fatty acids (REACTOME)	*INS*	rs4648808	11	1.07E-03
	*STXBP1*	rs1870516	9	5.80E-03
	*PLCB3*	rs1908490	11	4.02E-03
	*GNAQ*	rs7539775	9	2.30E-03
	*PLCB2*	rs2029490	15	4.11E-03

CHR, chromosome; KEGG, Kyoto Encyclopedia of Genes and Genomes; SNP, single nucleotide polymorphism.

## Discussion

In this study, a pathway-based approach was applied to a GWAS dataset of patients with PCOS. The study identified significant pathways involved in ovulation and insulin secretion, including oocyte meiosis and the regulation of insulin secretion by ACh and FFAs.

Pathway analysis is a post-GWAS analysis method that can be applied to further interpret GWAS results. Early pathway-based approaches employed raw genotype data for GSEA, which are not provided in all GWAS, and required intensive computational permutations [16]. To simplify the application of GSEA to GWAS data, pathway approaches using SNP *P*-values such as MAGENTA have been introduced; these approaches analyze the statistical power of GWAS by integrating the *P*-values for variant associations into gene scores [26]. Through MAGENTA pathway analysis, important pathways associated with triglycerides, low-density lipoprotein (LDL) cholesterol, BMI and type 2 diabetes can be identified [26, 27].

In the present study, oocyte meiosis was identified as the top-ranking biological pathway associated with PCOS. Oocyte quality, maturation and fertilization are affected by factors such as hyperandrogenemia and insulin resistance, which are important phenotypes of PCOS and can lead to premature follicular luteinization and anovulation [28, 29]. The regulation of insulin secretion by ACh was another top-ranking biological pathway associated with PCOS. Pancreatic ß-cells are regulated by various hormones and neurotransmitters; ACh is an important neurotransmitter that is released by intrapancreatic nerve endings and promotes glucose-stimulated insulin secretion through muscarinic ACh receptors [30]. Variation in this biological pathway could result in abnormal insulin regulation and glucose intolerance, which are important phenotypes of PCOS. The biological pathway related to the regulation of insulin secretion by FFAs was also associated with PCOS. Chronic FFA exposure can have a detrimental effect on insulin secretion and ß-cell function, with elevated FFA levels enhancing hepatic gluconeogenesis and insulin resistance in the liver and peripheral tissues [31]. In addition, obesity can increase fat deposition in islet cells, leading to insulin resistance and hyperinsulinemia, which are important metabolic features of PCOS [32].

*INS* was observed in all three top ranking pathways associated with PCOS. Previous studies showed an association of this gene with insulin resistance, obesity and type 2 diabetes through variation of the VNTR (variable number of tandem repeats) locus at class III allele [33–35]. *INS* was also associated with anovulation in PCOS, although there are conflicting studies [36–38]. These inconsistencies could be due to different diagnostic criteria used for PCOS, as well as different study groups or ethnicities. In our study, we used the NIH criteria for PCOS, which is a strict diagnostic method compared to Rotterdam or Androgen Excess Society criteria [21]. Severe metabolic abnormalities are seen in this group; studies show worse phenotypes for metabolic profiles and higher insulin resistance compared to non-NIH groups [2, 39, 40]. More studies on NIH-PCOS groups will be needed to further elucidate the association between *INS* and PCOS.

From the genes identified in the pathway of regulation of insulin secretion by acetylcholine and FFA, *GNAQ*, a Gq protein encoding gene, is a known candidate gene of PCOS that mediates the insulin induced translocation of GLUT4 in adipocytes and is associated with insulin resistance and obesity in PCOS [41]. Other genes such as *STXBP1*, *PLCB2* and *PLCB3* have not been identified in PCOS yet. However, published studies have demonstrated abnormal expression in these genes, leading to abnormal insulin secretion and disordered glucose homeostasis [42, 43].

Calcium signaling pathway might have an association with androgen excess. Calcium is crucial in gonadotropin secretion, and studies have shown that calcium signaling is affected by androgen levels [44, 45]. *LHCGR* was identified in this pathway, which is a known susceptibility loci of PCOS discovered through GWAS, having an association with hyperandrogenism [10, 46, 47]. Other genes such as *EGFR* and *PLCZ1* were also observed in this pathway. Abnormal expression of *EGFR* was related with oocyte incompetence in PCOS women [48]. P*LCZ1* is expressed in sperm, and variations of this gene lead to low fertilization and male infertility [49, 50]. Variation in these genes could be the cause leading to androgen excess in PCOS, although extensive studies proving this association are necessary.

Although there is a lack of studies on the genes identified in the biological pathways of oocyte meiosis and PCOS, many genes have been studied in other human diseases. Mutation of *SMC3* is related with Cornelia de Lange syndrome, characterized by features such as growth and mental retardation with abnormal limb formation, and associated with the development of atopic asthma and myeloid neoplasms [51–55]. Inactivation of *PPP1CB* caused chronic lymphocytic leukemia, whereas *CCNE2* was related to the development of non-small cell lung cancer and breast cancer [56–58]. Mutations in the *PP2A* regulatory subunit B family of genes resulted in features associated with overgrowth, and because it is an important gene in the phosphorylation of tau protein, which is crucial in neurofibrillary tangle formation, it could lead to Alzheimer’s disease [59–61].

We used a pathway-based approach to identify multiple biological pathways or gene sets that are involved in the pathogenesis of PCOS. To our knowledge, this was the first GWAS dataset-based pathway analysis study to be conducted for PCOS. One of the strengths of this study is that the subjects were accurately selected, and homogenous PCOS groups were recruited using well-defined diagnostic criteria. Although the identified pathways did not show an FDR of less than 0.05, significant pathways associated with ovulation and insulin secretion were discovered at an FDR of less than 0.2. Because ovulatory dysfunction and abnormal insulin secretion are major features of PCOS, the biological pathways identified in this study may be important. However, validation of these pathways using other pathway approaches will be necessary.

There are some limitations of this study. First, the number of women with PCOS included in the GWAS dataset is relatively small. Second, the pathway analysis tools applied in the study are biased toward detecting well-defined pathways. However, the majority of the genes in the genome are relatively unknown, and their biological function still needs to be established. Third, our study is confined to Korean women only. Because different phenotypes of PCOS are seen in women with different ethnic backgrounds, our results may not be generalizable to other ethnic groups. However, genes such as *LHCGR* have been identified as susceptibility loci in Han Chinese, Hui Chinese and Egyptian populations [10, 57, 62]. Therefore, similar biological pathways and genes may be found in these ethnicities, although pathway analysis will be required.

In conclusion, by applying pathway analysis to a GWAS dataset for PCOS, significant biological pathways and genes associated with ovulation and insulin secretion were identified. Our results may contribute to understanding the mechanisms underlying PCOS.

## Supporting Information

S1 Table Significant genes within other biological pathways or gene sets associated with polycystic ovary syndrome. (DOC)
