# Supplementary material for: Pathway Analysis Based on a Genome-Wide Association Study of Polycystic Ovary Syndrome
Source: PLoS One. 2015 Aug 26;10(8):e0136609. doi: 10.1371/journal.pone.0136609 (PMC4550465; doi:10.1371/journal.pone.0136609)
Supplement: S1 Table — (DOC) [file pone.0136609.s001.doc]

**S1 Table Significant genes within other biological pathways or gene sets associated with polycystic ovary syndrome.**

| **Biological pathway or gene set**  **(database)** | **Genes**  **(*P* <0.05)** | **Best SNP** | **CHR** | **SNP**  ***P-*value** |
| --- | --- | --- | --- | --- |
| Neural tube closure  (GO Term) | *APAF1* | rs1213975 | 12 | 4.34E-03 |
|  | *PTCH1* | rs2746064 | 9 | 7.58E-03 |
|  | *VANGL2* | rs707593 | 1 | 7.05E-03 |
| Other kinase  (PANTHER) | *ITGB1BP3* | rs2864230 | 19 | 9.03E-04 |
|  | *MORN1* | rs4018608 | 1 | 4.26E-04 |
|  | *CKMT1B* | rs1193171 | 15 | 9.57E-03 |
|  | *CHKA* | rs4129058 | 11 | 6.49E-03 |
| Calcium signaling pathway  (KEGG) | *LHCGR* | rs7549631 | 2 | 1.30E-04 |
|  | *AVPR1A* | rs3842878 | 12 | 6.04E-04 |
|  | *PRKCG* | rs1090983 | 19 | 3.41E-03 |
|  | *CACNAL1I* | rs3001336 | 22 | 1.48E-03 |
|  | *CCKBR* | rs2645066 | 11 | 1.32E-03 |
|  | *P2RX1* | rs903901 | 17 | 1.43E-03 |
|  | *PLCB3* | rs1908490 | 11 | 4.02E-03 |
|  | *HTR7* | rs1212015 | 10 | 4.47E-03 |
|  | *PLCZ1* | rs1075339 | 12 | 5.55E-03 |
|  | *MYLK* | rs1682302 | 3 | 2.46E-03 |
|  | *GNAQ* | rs7539775 | 9 | 2.30E-03 |
|  | *EGFR* | rs2493278 | 7 | 2.66E-03 |
|  | *CHRM2* | rs1684080 | 7 | 3.75E-03 |
|  | *PLCB2* | rs2029490 | 15 | 4.11E-03 |
| Acyltransferase  (PANTHER) | *AGPAT2* | rs1107685 | 9 | 1.65E-03 |
|  | *CHAT* | rs2864230 | 10 | 9.33E-04 |
|  | *GGTLA4* | rs882430 | 20 | 2.59E-03 |
|  | *EPB42* | rs2869401 | 15 | 4.73E-03 |
|  | *HIBCH* | rs2993510 | 2 | 2.14E-03 |
|  | *ELOVL5* | rs1211971 | 6 | 2.84E-03 |
|  | *AGPAT5* | rs2840534 | 8 | 1.51E-03 |
|  | *ME1* | rs7552331 | 6 | 2.59E-03 |
|  | *TGM7* | rs6663290 | 15 | 6.91E-03 |
|  | *DHDDS* | rs1202991 | 1 | 4.49E-03 |
|  | *FASN* | rs3753702 | 17 | 5.22E-03 |
|  | *LPCAT1* | rs7539775 | 5 | 2.31E-03 |
|  | *DLST* | rs2275909 | 14 | 8.50E-03 |
|  | *HHAT* | rs2477777 | 1 | 5.64E-04 |
| Negative regulation of osteoclast differentiation  (GO Term) | *INPP5D* | rs2340582 | 2 | 7.25E-05 |
|  | *TLR4* | rs2376804 | 9 | 9.63E-04 |
|  | *CTNNB1* | rs1256705 | 3 | 5.47E-03 |
| Cytoskeletal protein binding (GO Term) | *FRMD3* | rs7526076 | 9 | 1.68E-04 |
| Developmental processes  (PANTHER) | *EYA1* | rs4074137 | 8 | 2.05E-04 |
|  | *BCL11B* | rs1090718 | 14 | 2.37E-04 |
|  | *ID2* | rs2993480 | 2 | 2.15E-03 |
|  | *TLR4* | rs2376804 | 9 | 9.63E-04 |
|  | *POGK* | rs1091009 | 1 | 1.76E-03 |
|  | *LASS4* | rs12184 | 19 | 9.93E-04 |
|  | *SCML4* | rs1716066 | 6 | 5.36E-04 |
|  | *IGF2* | rs4648808 | 11 | 1.07E-03 |
|  | *NR2C2* | rs1553288 | 3 | 2.39E-03 |
|  | *HS3ST3A1* | rs2503701 | 17 | 1.14E-03 |
|  | *TSKU* | rs6424068 | 11 | 2.30E-03 |
|  | *BCL11A* | rs7539511 | 2 | 2.01E-03 |
|  | *POU2F2* | rs1126067 | 19 | 7.01E-03 |
|  | *MIA3* | rs1817914 | 1 | 4.16E-03 |
|  | *ZMYND12* | rs2821040 | 1 | 2.79E-03 |
|  | *DPF3* | rs7525092 | 14 | 8.23E-04 |
|  | *POGZ* | rs1710424 | 1 | 4.68E-03 |
|  | *HS3ST6* | rs7542208 | 16 | 3.83E-03 |
|  | *DYNLL1* | rs1079914 | 12 | 5.73E-03 |
|  | *TLE1* | rs2840534 | 9 | 1.51E-03 |
|  | *ZW10* | rs1079640 | 11 | 6.72E-03 |
|  | *ETV1* | rs7516150 | 7 | 2.58E-03 |
|  | *PAPPA* | rs4648636 | 9 | 1.61E-03 |
|  | *PMP22* | rs1075339 | 17 | 5.56E-03 |
|  | *CORIN* | rs7543737 | 4 | 2.19E-03 |
|  | *DLGAP2* | rs1159019 | 8 | 1.80E-03 |
|  | *E2F4* | rs2359908 | 16 | 1.71E-02 |
|  | *PLXNC1* | rs1090984 | 12 | 2.03E-03 |
|  | *WNT1* | rs912988 | 12 | 6.39E-03 |
|  | *TLE3* | rs625079 | 15 | 4.28E-03 |

CHR, chromosome; GO, gene ontology; KEGG, Kyoto Encyclopedia of Genes and Genomes; PANTHER, Protein Analysis Through Evolutionary Relationships; SNP, single nucleotide polymorphism.
